# Human Movement Is Both Diffusive and Directed

**DOI:** 10.1371/journal.pone.0037754

**Published:** 2012-05-30

**Authors:** Mark Padgham

**Affiliations:** Department of Geography and Planning, The University of New England, Armidale, Australia; Universidad Carlos III de Madrid, Spain

## Abstract

Understanding the influence of the built environment on human movement requires quantifying spatial structure in a general sense. Because of the difficulty of this task, studies of movement dynamics often ignore spatial heterogeneity and treat movement through journey lengths or distances alone. This study analyses public bicycle data from central London to reveal that, although journey distances, directions, and frequencies of occurrence are spatially variable, their relative spatial patterns remain largely constant, suggesting the influence of a fixed spatial template. A method is presented to describe this underlying space in terms of the relative orientation of movements toward, away from, and around locations of geographical or cultural significance. This produces two fields: one of convergence and one of divergence, which are able to accurately reconstruct the observed spatial variations in movement. These two fields also reveal categorical distinctions between shorter journeys merely serving diffusion away from significant locations, and longer journeys intentionally serving transport between spatially distinct centres of collective importance. Collective patterns of human movement are thus revealed to arise from a combination of both diffusive and directed movement, with aggregate statistics such as mean travel distances primarily determined by relative numbers of these two kinds of journeys.

## Introduction

Understanding the movement patterns of humans or other animals requires understanding the processes that generate variations in both time and space. Examining spatial patterns of movement is often more difficult in humans than in other animals, because of the dependence of human movement on our own complexly constructed environments [1], [2]; because of a tendency for sedentary humans (and some primates; [3]) to start and end journeys from home [4], [5]; and because of pragmatic difficulties associated with the close monitoring of human movement.

Although these latter difficulties can be circumvented through recent technology enabling detailed yet anonymous tracking [4], [6]–[11], geographical dependence of movement patterns requires generalised studies to focus either on abstracted spatial environments such as subway tunnels [12], footpaths [13], or automobile expressways [14], or on abstracted movement variables such as journey distances or durations alone [4], [9], [15], or relative directions [6]. This necessary abstraction effectively removes any explicit dependence of movement patterns on actual journey origins and destinations, as well as on actual directions of movement [16], [17].

Human behaviour is nevertheless very strongly geographically bound [18], [19], and most movements must be presumed to be motivated by a desire to travel between particular origins and destinations. While individual movements can be framed relative to fixed home points [4], collective movement must also be presumed to be directed to and from centres of shared importance. Although spatial variations in collective human movement [6], [20]–[22] are influenced by environmental structure [1], [2], understanding general relationships requires a means of quantifying spatial structure in a general sense.

This quantification can not, however, simply be of geographic structure alone [23], because collective agreement in projecting cultural values onto landscapes initiates and continues historical processes that reconfigure geography to reflect human culture, and that also reconfigure human culture to reflect geography. It is thus not geography alone that influences collective human movement, but also shared cultural understandings of geography. The present work demonstrates a technique to quantify the structure of such a ‘cultural geography’ [24] in terms of relative degrees of movement oriented both towards and away from each point [25].

The cultural and geographical space considered here is the centre of London, U.K., as revealed through analyses of public data [26], [27] from the Transport for London Barclay's Bicycle Hire Scheme, detailing origins and destinations of 1,425,884 trips taken throughout the city over 97 days. The article begins with analyses of the spatial and temporal patterns of movement [11], [21], [22], [28], following which the method is described for analysing vector trajectories to reveal locations of cultural and geographical significance towards, away from, or around which movement circles. Analyses conclude by relating the resultant fields of relative convergent and divergent movement to the observed spatial variations in movement variables, following which are discussed the general implications for understanding how cultural geography influences human movement.

## Results

### Spatial variations in movement

**Figure 1:**
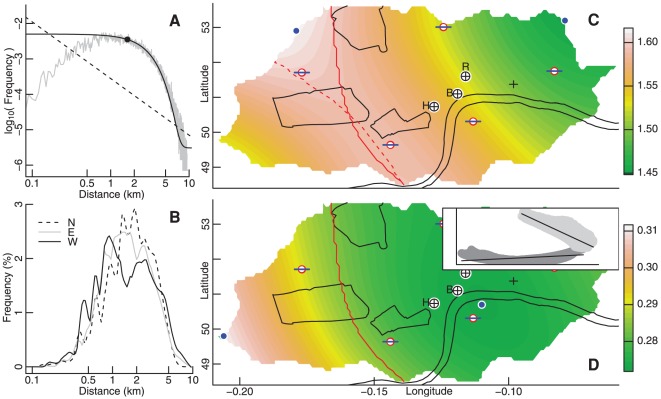
Frequency distributions and maps of ride distances. (A) Frequency distribution of ride distances (grey line). Black dot shows median distance (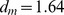 km), while solid black line shows least-squared error Gaussian curve fitted to the portion 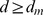, which was 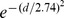. Dashed black line shows typical power-law distribution of 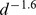. (B) Distance distributions within 2 km of northern (at −0.15

**Figure 2:**
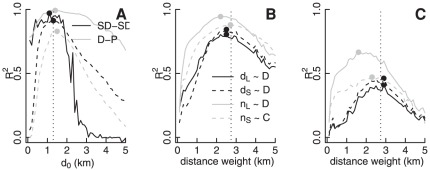
Effects of distance weighting and short–long division. (A) Correlations between observed and reconstructed SDs (‘SD–SD’), and between observed distances and proportion of long trips (‘D–P’), as a function of the distance separating short from long trips. Correlations were calculated both spatially (through aggregating data across the whole day, and comparing stations; shown by dashed lines) and temporally (through aggregating data across all stations, and comparing the 18 hours of the day; shown by solid lines). Vertical line indicates the division used in all analyses of 1.3 km. (B) 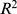 correlations between distances and numbers of short (n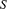 and d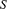) and long (n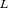 and d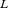) trips, and convergence (C) and divergence (D) fields, as functions of the distance weight used to project data onto the regular grid. Vertical line indicates chosen value of 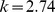 km. (C) Repeat of panel (B), but using radial divergence and convergence rather than the full measures (see Methods).

The bicycle docking stations from which data were collected are irregularly distributed throughout the city, with numbers of both bicycles, and of rides originating from or travelling to each of the 351 stations analysed here, decreasing linearly with distance from the city's centre (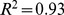 in both cases). Because analyses of spatial pattern require sampling to be regular, the bias introduced in patterns extracted directly from these spatially irregular data was removed through projecting all quantities aggregated at each station onto a spatially regular grid of 351 points spanning the same area as the bicycle network, with contributions from each station weighted by distance to each grid point according to the observed frequency distribution of ride distances (Fig. 1A; effects of different weighting functions are illustrated in Figs. 2B, C; see Methods). This projection is a form of spatial smoothing, the effects of which were revealed through also conducting analyses with the spatially irregular data collected at each station (see Table 1, below).

W), eastern, and western extremities of the bicycle system, with linear frequency scale. Distributions smoothed to aid visual display. (C, D) Maps of (C) mean and (D) SD ride distance in km and log km, respectively, plotted against longitude (ordinate in degrees east; spanning 10.5 km) and latitudes between 51.49 and 51.53

N (abscissa; spanning 5.8 km), both in 0.01 increments. Maps show the River Thames; St. Paul's (black cross); and the train stations (red and blue London Tube symbols, clockwise from east) of Liverpool Street, Waterloo, Victoria, Paddington, and King's Cross. Black outlines clockwise from top depict Regent's Park, St James/Green Park/Buckingham Palace Gardens, and Hyde Park. Circled crosses denote: the official centre of London (H, in Trafalgar Square); the centre of the network of bicycle stations (B, on the Strand); and of all rides actually taken (R; in Holborn). Blue dots mark positions of minima and maxima within each panel. Inset in (D) shows relationship between mean and SD ride distances respectively plotted on horizontal and vertical axes, with scales as in panels (C) and (D). Minimal error bi-linear regression as indicated (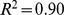) precisely divides east from west either side of the solid red line shown in both panels. Dashed red line in (C) traces ridge of maximal mean distance.

**Table 1:** Spatial and temporal correlations between observed and modelled movement variables.

	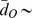				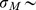	
	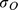	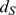	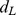	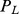	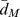	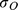
Time(I)	−0.54	0.76	0.79	0.99	−0.58	0.95
Time(R)	−0.73	0.75	0.85	0.97	−0.44	0.79
Space(I)	−0.72†	0.23	0.12	0.83	−0.72†	0.91
Space(R)	−0.90†	−0.44	0.51	0.79	−0.81†	0.89

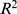
 correlation coefficients between observed (O) and modelled (M) mean (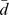) and SD (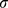) distances, as well as mean short (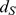) and long (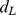) distances, and the relative proportion of long trips (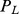). Relationships are given between irregular spatial averages aggregated at each station (‘I’), and after projection onto a spatially regular grid (‘R’).

† Spatial 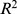-values for 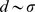 are from bi-linear regressions like that illustrated in Fig. 1D, with signs reflecting those of corresponding full linear regressions.

In contrast to the centrality of the bicycle system, spatial distributions of both mean and standard deviation (SD) ride distances (respectively on linear scales as 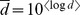 and on logarithmic scales as 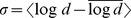) were distinctly non-centralised, and distinctly different from one another (Figs. 1C, D). The relationship between mean and SD trip lengths was bifurcated (inset of Fig. 1D), with the two portions of the illustrated bi-linear regression neatly delineating London east from west.

This spatially variable relationship between 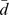 and 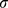 implies that the fields of Figs. 1C and D can not have been produced by any single, spatially invariant process [29]. Rather, as suggested by the accuracy of the bi-linear model, these spatial distributions—and thus also the aggregate distribution of Fig. 1A—are likely to have been generated by two distinct yet related processes, as further indicated by the bi-modal distribution in the west of the city (Fig. 1B). This negative spatial relationship between 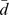 and 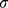 was also reflected in time, with the correlation between hourly values aggregated from all stations also strongly negative (see Table 1, below).

### Lengths and numbers of journeys

The distribution of rides in the west of the city suggests a distinction between ‘short’ and ‘long’ trips (Fig. 1B). The extent to which such a distinction might underlie the bifurcated relationship between mean and SD trip lengths was examined by separating rides either side of a variable distance, 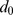, to enable separate analyses of both short and long trips in terms of lengths, respectively denoted 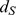 and 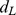, and numbers, denoted 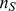 and .

These values were then recombined to produce a single probability distribution for trips of length 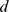 as 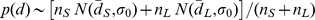, where 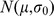 denotes a normal probability distribution with mean 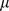 and SD 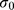, with the latter held constant throughout (and all results invariant for all values of 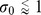 km).

Across a range of values of 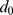, the ability of this categorisation of short and long journeys to reconstruct observed variations in SD distances was tested both in space through comparing observed and reconstructed distributions at each station, and in time through comparing hourly distributions of rides aggregated across all stations, with 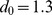 and 1.1 km yielding the respective minimal errors (and with the former value used throughout; Fig. 2A). Reconstruction was very accurate (Table 1), demonstrating that observed temporal and spatial variations in SD need not reflect a single process with a complex and variable SD, but can simply reflect variations in the combination of two processes of equal and constant SD representing short and long journeys.

**Figure 3:**
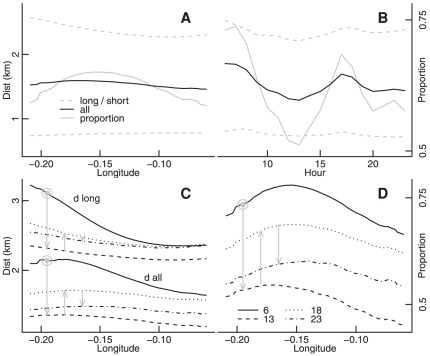
Spatial and temporal variations in movement variables. Mean lengths of long (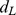), short (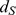), and all (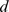) trips as a function of (A) longitude and (B) time of day, both on same vertical scale shown on left of (A). Both panels also show the proportion of long rides on same scale shown on right of (B). (C) Variations in spatial patterns of 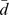 and 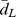 throughout the day, with temporal progression indicated by grey arrows connecting from left to right the hours 6:00 to 13:00 (decreasing); 13:00 to 18:00 (increasing); and 18:00 to 23:00 (again decreasing). (D) As for (C), but for the proportion of long trips.

Numbers of short and long trips were positively correlated (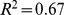), with decreases in trip numbers associated with decreases in the lengths of short journeys (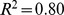), yet with increases in the lengths of long journeys (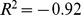), suggesting that the separation of the two peaks of Fig. 1B towards the west of the city reflects a generally linear process. Furthermore, variations in mean ride distances were most strongly correlated in both time and space with the proportion of long rides, 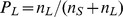, rather than with actual distances of either short or long rides (Table 1). Spatial variations in 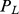 (Fig. 3A) clearly revealed the cause of both the delineation of east from west London, and of the increase in SD trip lengths in the far east (Fig. 1D). Moving westward towards the delineation, both lengths and proportions of long trips increased. Lengths of long trips continued to increase west of the delineation, yet their proportion decreased, producing an overall decrease in mean trip lengths.

**Figure 4:**
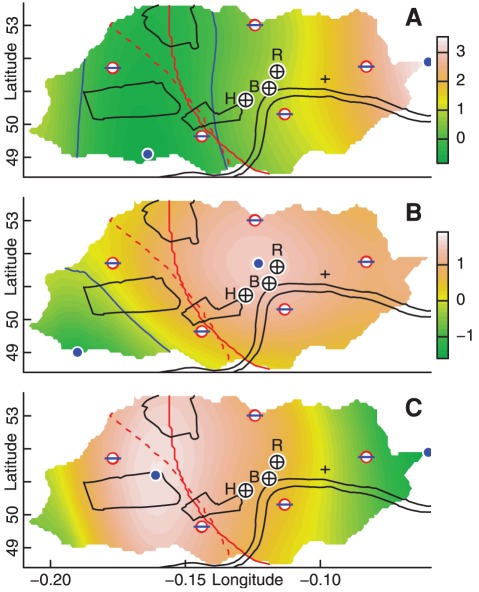
Convergence and divergence maps. Maps of net vector (A) convergence and (B) divergence, constructed as for Figs. 1C and D. Scales represent anomalies above expected values, in units of 1,000 rider km/day, with blue dots marking locations of maxima and minima. Solid red lines delineate the two bi-linear portions relating 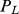 to 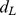; dashed red lines trace ridge of maximal 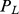 (directly comparable to equivalent lines in Fig. 1C; compare 1-D version of Fig. 3A); and solid blue lines delineate regions of positive from negative net flux. (C) Mean trip distance reconstructed from individual regressions of 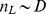 and 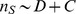.

These spatial patterns remained qualitatively very similar throughout the day, with temporal variations (Fig. 3B) reflecting synchronous shifts in 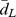, 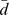, and 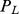 across the city (Figs. 3C, D). Temporal variations in mean ride distances were predominantly explained (84%) by shifts in the scale of Fig. 1C, with only 16% explained by relative changes in the spatial distribution, a value commensurate with previous results from Shenzen, China (of 26%; [11]). In two dimensions, the contour of maximal 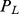 (see Fig. 4, below) very accurately reproduced the ridge of maximal ride distances (Fig. 1C), while the spatial delineation obtained from the bi-linear regression between 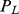 and 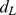 (also in Fig. 4) very accurately reproduced that between mean and SD distances (Figs. 1C, D).

Thus arises one fundamental contribution of the present work, in the demonstration that patterns of collective movement do not arise from one single process. Rather, in contrast to statistical descriptions of movement as a singular, aggregate phenomenon (for example, [9]–[11], [15], [21]), these analyses demonstrate that the distinct spatial patterns of mean and SD ride distances observed here (Figs. 1C, D) minimally requires the combination of two homoscedastic processes distinguished by spatial variation in mean distances. Moreover, the fact that temporal variations in these two process maintain the same relative spatial structure across the city (in terms both of mean distances and proportions of long to short trips; Fig. 3) strongly suggests the influence of a fixed geographical template, as now further explored.

### Relationships with underlying spatial structure

The fixed spatial patterns discerned above must reflect collective movement oriented to, and strongly influenced by, a spatially heterogeneous landscape. It is, however, not just spatial heterogeneity—or, in general, geography—itself that influences movement, but also cultural values as collectively projected onto geographical structure. The former can be directly and objectively quantified; the latter can not. These dual influences on movement dynamics were examined here through quantifying the extent to which locations of cultural or geographical significance (hereafter, ‘significant locations’) cause movement to be oriented in some structured way relative to the random orientation expected in an homogeneous landscape [25]. Because significant locations likely act both as point sources producing a ‘social force’ [30] effecting radial movement, and also as centres around which movement circles, the structuring of movement was quantified here in terms of total numbers times distances of rides (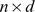), distinguished only by whether overall orientation was towards (convergence, 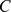) or away from (divergence, 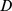) each location (see Methods). Moreover, because significant locations are likely to engender equally greater convergence and divergence, the two fields were considered separately here, in contrast to conventional calculations of net divergence (that is, divergence minus convergence). Significant locations will produce increases in one or both of these fields that extend across some finite spatial range.

The resultant fields were predominantly structured by greater absolute cycling activity in the city's centre, and mirrored the strong centrality described above of both London itself and of the bicycle system, analogous to most other work on movement in urban spaces [21], [22], [28]. However, the relative temporal invariance of the spatial structures of Fig. 3 demonstrates that the cultural and geographical landscape studied here (and likely that in [11]) influences movement independent of absolute numbers, precisely as would be required for a landscape to influence individual journeys. These flux fields of convergence and divergence therefore must also be calculated in a relative sense.

Flux fields vary in time or space through variations in the degree of orientation towards significant locations. The necessary rescaling of these fields was achieved here relative to expected orientations calculated from an equivalent set of rides simulated to be dependent only upon the intrinsic properties of the bicycle network, yet randomly oriented in space. Differences between flux patterns arising from the observed and simulated rides thus quantify the relative degree of intentional orientation towards, away from, or around, significant locations. To ensure adequate spatial sampling, 100 times as many rides (thus totalling 140 million) were simulated as actually observed, with resultant fluxes divided by 100 for subsequent comparison (see Methods).

The simulated numbers of trips, 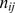, between each pair of stations, 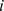 and 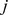, can be used to generate a spatial distribution of expected probabilities, 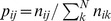, where 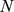 is the number of bicycle docking stations. The probability that 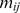 trips are actually observed can then be calculated as [31],
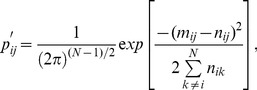
(1)such that greater differences between the observed and expected distributions will produce lower probabilities. Conversely, some appropriately inverted form of these probabilities will provide a measure of the relative extent to which fluxes of cyclists are spatially non-random because of movement towards, away from, or around significant locations. However, this equation describes the probabilities of moving from one single node, 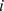, and the required probability distribution must be obtained by additionally summing over all nodes, 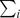. The denominator of the exponential in this case simply equals the total number of rides, 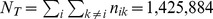, and the logarithm of Eq. 1 then gives,
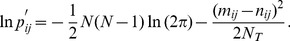
(2)Re-writing this as,
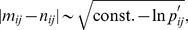
(3)demonstrates that absolute differences between observed and expected numbers of trips, 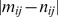, scale monotonically, albeit non-linearly, with the desired probability in inverted form. Relative convergence and divergence fields can therefore be calculated directly from absolute differences between observed and expected numbers of rides. Moreover, Eq. 1 merely describes the probability of a given difference in the spatial distribution of rides, regardless of whether differences are positive (more rides) or negative (less rides). The signs of these differences are, however, meaningful in this context, and so fields were calculated from the full signed differences, with negative values indicating lower than expected fluxes (Figs. 4A, B).

In an homogeneous space with uniformly distributed bicycle docking stations, convergence and divergence will be equal yet each will be individually greater than zero. The relative fields simply remove these non-zero components expected with random orientation, such that non-zero values will only arise from collective orientation towards, away from, or around significant locations. In the absence of particular hypotheses, movement must be generally presumed randomly oriented; these relative fields must be expected to be devoid of structure; and no relationships will be expected with the spatial patterns of movement variables observed above. In contrast to this ‘null’ hypothesis, the strong relationships observed here (Table 2) must reflect non-random orientation of movement to the underlying cultural and geographical landscape.

**Table 2:** Correlations between movement variables and flux fields.

Variables	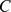	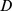	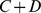	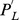
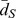	0.43	**0.87**	0.82	
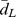	−0.46	−0.78	**−0.81**	
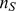	0.74	0.58	**0.93**	
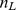	0.28	**0.94**	0.70	
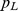	−0.63			**0.74**
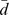	**−0.83**		−0.72	0.68
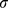		**−0.88**	−0.64	

Strengths of relationship (as 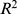 correlation coefficients) between convergence (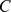), divergence (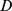), total flux (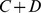), and 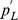 as modelled from 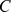 and 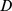 and explained in text; and lengths and numbers of short and long trips, the proportion of long trips, and mean (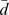) and SD (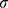) distance. Bold font indicates maximal correlation for each movement variable. Values of 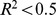 are not shown.

Reflecting the above relationships between trip numbers and distances, lengths of short trips (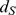) increased with increasing flux (most strongly with divergence), while both mean overall and long-trip distances decreased (with the strongest relationships being 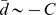 and 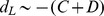; Table 2). The relationships between fluxes and numbers of trips were then used to reconstruct 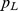 based on the positive linear relationships of 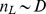 and 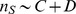 (Table 2), with the resultant field of 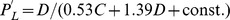 (Fig. 4C) accurately reproducing the spatial field of mean ride distances.

Importantly, while spatial distributions of distances and numbers of trips remained qualitatively similar throughout the day, as previously described (Figs. 3C, D), flux fields changed considerably (Figs. S1 and S2), with only those aggregated across the entire day accurately reproducing spatial variations in movement dynamics. This suggests that the two flux fields of daily convergence and divergence accurately captured the spatially fixed aspects of the city's cultural geography.

### Angular orientation

Although measures of convergence and divergence generated by random movement through an infinite, homogeneous environment will be equal, each will be non-zero without representing any actual directional preference. Similarly, although the flux fields calculated as described above quantify the relative degree of non-random movement towards and away from each location, they nevertheless do not represent any particular angular orientation. A non-zero measure of adjusted convergence or divergence simply means that convergent or divergent movement towards or away from a given point exceeds that expected with random orientation, regardless of whether or not that convergence or divergence is oriented along any particular direction.

The extent to which travel is actually oriented both away from gradients of divergence and towards gradients of convergence thus provides an independent measure of the extent to which travel is actually oriented to the local cultural or geographical features as represented by the spatial structure of the flux fields. The non-random structure of the bicycle system itself, however, generates preferred directions of travel, predominantly towards and away from the city's centre where there are more bicycle stations. The directly observed angular orientations were therefore adjusted by subtracting the net movement vectors generated by the simulated set of 140 million randomly-oriented rides (see Methods).

**Figure 5:**
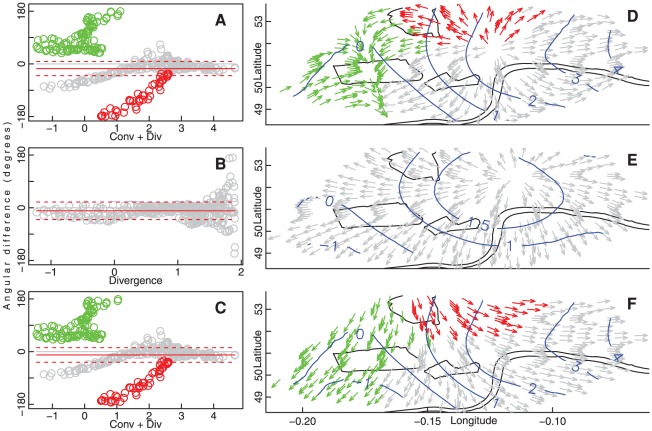
Angular differences between movement vectors and flux gradients. (A–C) Angular differences between net movement vectors adjusted for system structure and expected vectors following gradients of vector fields against which movement vectors were most strongly aligned, plotted against respective field strengths for (A) all, (B) short, and (C) long trips. Red and green points in (A, C) identify outlying groups excluded from calculations of mean angular differences. Anti-clockwise angular differences between observed and expected angles are positive; clockwise are negative. Flux values on abscissa are in units of 1,000 ridden kilometres per day, as in Fig. 4. Red lines show mean (solid) 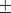 SD (dashed) angular differences (excluding red and green circled points). (D–F) Net movement vectors of (D) all, (E) short, and (F) long trips, with colours in (D, F) corresponding to outlying regions identified in (A, C). Blue lines trace contours of corresponding flux fields (Fig. 4, and abscissae of corresponding panels A–C).

Angular differences were then calculated between these net adjusted movement vectors and maximal gradient vectors away from divergence and towards convergence, combined according to the relative strengths of the fields. Alignment with individual fields was also examined, to identify the field (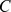, 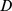, or 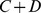) with which movement was most strongly aligned. Net adjusted movement vectors still generally radiated out from the city's centre (Fig. 5D), and were most strongly aligned to gradients of the combined (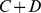) field, with a mean 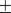 SD angular difference of 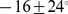 (Fig. 5A). Orientations of net movement vectors constructed from long trips only were similarly aligned to the combined field, with angular differences of 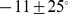 (Figs. 5C, F). In contrast to both of these results for which alignment improved with increasing field strength (Figs. 5A, C), short trips were most strongly aligned with divergence alone (Fig. 5E), with angular differences of 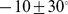 (Fig. 5B) discernibly increasing with increasing flux.

### Relationships with larger scale transport

The strong radial divergence observed in particular for short trips (Fig. 5E) obviously requires cyclists to first travel to the city centre before cycling outward. These patterns of convergence and divergence must therefore be considered with regard to the use of other modes of larger-scale transport to and from the city centre, predominant among which is the underground railway, or ‘Tube.’

**Figure 6:**
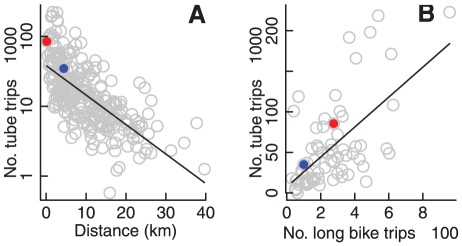
Relationships with underground rail system. (A) Relationship between numbers of passengers per day entering or leaving underground (‘Tube’) railway stations, and the distance of those stations from the global centre of divergence at 0.123

Correlating numbers of passengers entering and exiting the Tube [32] with the distance of each Tube station from the global centre of divergence revealed that this divergent centre also represents the approximate centre of activity of the underground railway system (Fig. 6A; and see [22]). Numbers of passengers entering an exiting each Tube station were strongly correlated with numbers of long journeys starting or ending near those stations (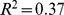; Fig. 6B), yet were only weakly related to numbers of short journeys (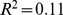).

W, 51.517

N. Regression line shown in black (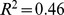). The red dot indicates passenger numbers at the station closest to the centre of divergence (Holborn Station, 85,600 passengers per day), and the blue dot the equivalent for convergence (Whitechapel Station, 34,800 passengers per day). (B) Relationship between total daily numbers of long bike trips (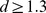 km) closest to each Tube station within the bicycle network area, and corresponding numbers of Tube passengers. Regression line shown again in black (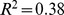), with coloured dots corresponding to those in panel (A).

## Discussion

Public bicycles greatly facilitate the dense permeation of large urban centres, and data from their usage offer unique opportunities to examine the influences of cultural and geographical spaces on human movement [21], [28]. In this study, spatial and temporal variations in numbers of rides had a greater influence on observed mean distances than did variations in individual journey distances. Because relationships between numbers and lengths of rides are almost universally inverted, only one of them—in this case, numbers of rides—will positively determine observed mean distances at any point in space or time, while the other—distances—will be negatively correlated as in Table 2, reflecting a secondary, indirect influence. Accurate understanding of human movement thus requires more than just aggregating individual journeys while ignoring (causes of) spatial heterogeneity in journey numbers. In fact, while temporal variations in journey numbers and distances were pronounced (Fig. 3B), 84% of this variation arose through changes in the relative scales of fixed spatial distributions (Figs. 3C, D) [11], with spatial distributions of mean travel distances primarily dependent upon the relative numbers of different kinds of trips, rather than on actual distances of individual trips.

Discernment of such apparently fixed spatial templates does not in itself, however, enable any explanation of their origin, and therefore nor any generalisation beyond the particular place of a given study. Attaining such generalisation requires relating variations in movement dynamics to the general properties of underlying cultural and geographical features, demanding a means of independently quantifying landscape structure in a general sense. The primary contribution of the present work is in providing precisely such a means, through constructing relative flux fields that capture only those components of movement that are non-randomly oriented towards, away from, or around significant locations. These flux fields provide a direct quantification of cultural or geographical significance that may be independently related to aggregate variations in journey distances, numbers, and directions.

Relationships between the flux fields and both short and long journeys enable further elucidation of differences between these two categories of journeys. The centralised field of divergence (Fig. 4B) indicates an even stronger tendency than expected under random orientation for travel to diverge from the centre, and very likely reflects the use of other modes of transport, notably including the Tube, to first converge towards this centre, before using bicycles to diverge away. Divergent movement was most apparent in the alignment of short journeys (Fig. 5E), the lengths of which also decreased with decreasing divergence. Their numbers were, however, most strongly related to total flux (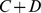), and were only weakly related to numbers of Tube passengers, suggesting that these numbers are directly determined by overall cultural or geographical significance, and that short trips represent effectively random, diffusive movement [33] away from regions of significance.

Relationships with long journeys were, in contrast, effectively reversed, such that trip numbers were most strongly related to divergence alone, while distances and orientations were determined by the combined fields of divergence and convergence (Figs. 5C, F). Orientations were, however, related to flux gradients only across the central, southern, and eastern portions of the city, with distinctly different patterns in the north and far west. While differences in the north can be readily interpreted through reference to the general, centrally divergent pattern of all rides (Fig. 5D), to reveal that long trips in this portion of the city differ by actually serving transport towards the divergent centre, long rides in the far west appear to reflect transport away from the city centre, yet in directions unrelated to divergence gradients.

However, the adjusted fields for both convergence and divergence decreased below zero in moving west from the city's centre (Figs. 4A, B). Rather than negative divergence implying convergence, it merely reflects a weaker tendency than expected for movement to be divergent. The orientation of rides with flux gradients thus ought to be generally weaker as relative flux fields decrease towards and then further below zero. Accordingly, as both convergence and divergence fields decreased towards the west of the city, so did the collective alignment of long trips with the gradients of these fields (Figs. 5C, F). (The aggregate southward trajectory apparent in the latter figure reflects a non-significant yet pervasive tendency of all trips to move an average of 41 m to the south.)

Extending beyond previous analyses of ‘polycentric’ urban structure [22], [34], these analyses effectively partition the city into three regions, with long trips collectively oriented to cultural or geographical features only within the largest portion comprising 60% of the city. Although orientations were not aligned to structures identified through the flux analyses within the remaining 40% of the city (indicated by green and red arrows in Fig. 5F), both numbers and distances nevertheless remained strongly related (Table 2).

Rather than these two categories of journeys representing regular movement to and from fixed ‘home’ points [3], and other movement [4], or any distinction between work- and non-work-related travel [28], the separation here of short from long journeys provided an effective way to distinguish between two categorically different kinds of journey. Shorter journeys serve diffusive movement [33] generally directed away from locations of cultural or geographical importance, while the relationship of their lengths with divergence suggests a ‘social force’ [30] type of effect by which greater divergent flux increases the lengths of these trips. These trips are accordingly interpreted, and referred to from hereon, as ‘diffusive’ trips. (Because of a continual supply of cyclists, the cycling system is open and diffusion is not able here to be diagnosed through its closed-system characteristic of distributional width increasing with the square root of time. Nevertheless, diffusion in both open and closed systems results in vector trajectories to be directed outward from points of supply, exactly as observed here in Fig. 5E.)

The second category represents longer journeys that are not just oriented away from centres of larger-scale divergence, but actively engage in the cultural geography of the city through also being oriented towards geographically distinct regions of convergence. These trips are also more directly connected to other modes of transport, and are interpreted, and referred to from hereon, as ‘directed’ trips. The contrasting properties of the two types of journeys are summarised in Table 3.

**Table 3:** Diffusive versus directed movement.

Variable	Diffusive	Directed
Length	short	long
Flux 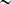 Length	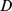	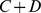
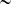 Orientation	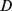	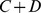
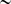 Number	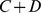	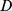
Tube 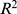	0.11	0.37

Properties of two classes of journey as distinguished both by lengths either side of 1.3 km, and by relationships between flux fields and journey lengths, orientations, and numbers. The strongest relationships with flux fields are indicated in each case, either as divergence alone (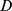), or total flux (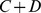). All relationships are positive, except the negative relationship between lengths of directed trips and 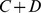 (see Table 2). Correlations with numbers of Tube passengers are also shown.

### General Implications

This study reveals two categorically different kinds of journeys, and the necessity of two distinct fields or ‘layers’ [35] to describe any single space of cultural geography. The functions of each of the two categories of journey were, however, only able to be inferred through reference to both fields, and the necessity of these two fields therefore can not merely reflect the distinction of two categories of journey. Rather, any description or model of spatial variations in movement dynamics is likely to require multiple spatial fields because of the hierarchical structure of transport systems. Movement within any urban area will be sustained by collective convergence from beyond as enabled by transport infrastructure at larger scales. Centres of divergence at any given hierarchical level are thus likely to be centres of convergence at higher levels (for a concrete illustration of convergence within the London Tube, see Fig. 6 of [22]). Subsequent movement will for many people simply diverge away from such larger-scale convergent centres, while others will continue to orient themselves toward places of collective significance. These two modes of movement represent two categorically distinct ways by which people engage with a given landscape and transport system, and this study demonstrates that the influence of spatial structure on movement can not be represented through a single spatial map, but rather requires the mapping of two distinct surfaces.

Moreover, individual humans generally move greater distances with probabilities or frequencies of occurrence, 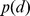, often decreasing according to a power law, 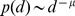, where 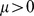 [4], [10], [22], [36]. However, there generally exists a minimal movement distance below which observations diverge from such power laws (commonly 5–10 km; [9], [15]). Because shorter journeys occur more frequently than longer journeys, studies examining the power-law distributions of movement distances neither represent nor reflect the majority of journeys. Power laws nevertheless often prove ‘surprisingly’ accurate at describing distributions of movement distances [15], suggesting they arise from a universal dynamic of movement [37].

While the small size of London's bicycle system prevents journeys of sufficient length to fit a power-law distribution here (Fig. 1A), the approximate agreement between the median trip distance of 1.64 km and the distance of 1.3 km dividing diffusive from directed journeys suggests that such a categorical distinction may also underlie the commonly observed failure of travel distances to adhere to power laws below a certain limit [9], [15]. Although such departure from power-law behaviour might be taken to suggest analogous departure from a ‘universal’ movement dynamic [37], the shorter, diffusive journeys observed here were in fact strongly regulated by cultural and geographical structure in their orientations, distances, and numbers.

Assuming that a bicycle system of unlimited size would indeed result in a power-law distribution of longer trips, then these results suggest that, rather than power law distributions arising through aggregation of individually diffusive—and therefore undirected—movement, as previously observed [38], they arise through directed orientation within heterogeneous environments [39]. However, modelling the processes generating such distributions requires assuming spatial structure to be also distributed according to a power law [39], effectively transferring the question of why power laws describe distributions of movement distances to why they describe distributions of spatial structure.

Repeating the present analyses over larger scales, and including other modes of transport, would offer an ideal way to examine whether or not spatial structure might be ultimately responsible for observed adherence of movement distributions to power laws. This work also provides an ideal basis from which to examine in more detail the nature of collective orientation towards and away from significant locations. In particular, such collective orientation could arise because of a single, culturally shared map of a geographical space (however approximate individual representations may be), or because individuals respond more to proximal cues that themselves are mediated by a larger structure. Extending from the present work to tease apart these different mechanisms of orientation [40]–[42] is likely to offer profound advances in understanding the influence of cultural geography on human movement.

## Materials and Methods

### Data used

The data were obtained from the Guardian newspaper's website [43], and extended across the 97 days from the first day of operation of the bicycle system on 30 July, until 3 November, 2010. Bicycles may only be taken between the hours of 06:00 and 23:59, with analyses extending across the 18 operational hours each day. There were a total of 1,425,884 trips starting and ending at different stations.

A number of trips started and ended at the same stations, likely because the data extended from the first day of operation of the bicycle system, and bicycles were taken for novelty alone, rather than for any purposefully directed travel. Relative numbers of these trips, however, decreased rapidly, from over 7% on the first day to under 3% after one month to around 1.5% at the end of the 97 days covered here. These trips were therefore excluded from all analyses, leaving the total of 1,425,884 trips starting and ending at different stations.

**Figure 7:**
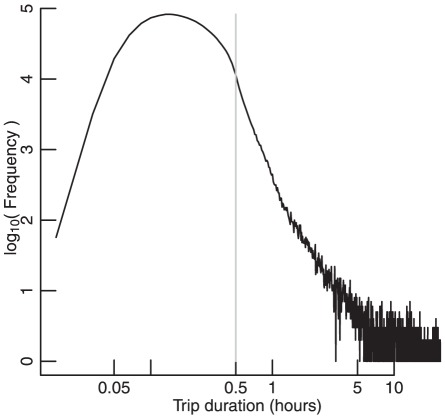
Frequency distribution of ride durations. Vertical line indicates 30 minutes, below which duration bicycle rental is free.

Use of the bicycles is free for the first 30 minutes, after which time users are charged according to the duration of a ride (according to the costs detailed at http://www.tfl.gov.uk/roadusers/cycling/14811.aspx). Although this financial incentive to restrict rides to less than 30 minutes may have influenced the observed distribution of ride durations (Fig. 7), a 30-minute trip nevertheless corresponds to 4.09 km in the analogous distribution of distances—well beyond the distance of 1.3 km that distinguished long from short trips. The primary results of this study are thus unlikely to have been affected by the extent to which ride durations may have been intentionally restricted to less than 30 minutes.

The number of stations was sufficient to ensure that all 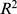 statistics reported in the main text were entirely significant (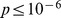), and significance values are therefore not explicitly stated.

### Spatial projection

Quantification of the spatial regulation of movement dynamics will only be unbiased to the extent that spatial sampling is regular. It was therefore necessary to project data from the spatially irregular bicycle docking stations onto a spatially regular grid. This projection combined the weighted effects of multiple stations at each point, and was therefore a form of spatial smoothing. Such smoothing is appropriate because any hypothesis that geography affects movement dynamics must consider geography as a continuum; in other words, must presume that movement near a particular point of cultural or geographical importance will be determined by distance to that point. This smoothing is equivalent to presuming that movement dynamics at a particular point can be estimated through combining independent (distance-weighted) contributions from the stations surrounding that point.

The regular grid was constructed from 351 points, the same number as of bicycle docking stations. The maps shown in the main text (Figs. 1, 4) were constructed on a grid of 5,510 points spaced at 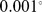 latitudinal and longitudinal intervals. The 351 points of the regular grid were spaced 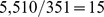 units of 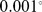 longitude apart, starting at a randomly selected grid box in the south east, and progressing in a raster scan pattern first east and then north.

### Distance weighting and the short–long distinction

The Gaussian weighting function of 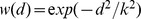 with 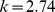 km was the least-error fit to the frequency distribution of trip distances beyond the median distance, as indicated in Fig. 1A. The effect of different values of 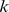 on the relationships between the divergence (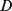) and convergence (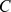) fields, and on both numbers and lengths of short and long trips, are illustrated in Fig. 2B, for which relationships with the single component (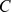 or 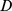) of greatest influence (Table 2) was analysed. All relationships were maximally strong, and of minimal error, very close to the value of 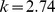 km.

The distinction between short and long trips was examined both in terms of the ability to reconstruct spatial and temporal variations in SD, and in the strength of relationship between trip distances and the proportion of long trips (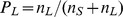). Values were compared as measured at each station, not as projected onto the spatially regular grid. In all cases, minimal errors were precisely coincident with maximal correlations, and all were very close to the chosen value of 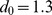 km (Fig. 2B).

### Calculation of convergence and divergence

Divergence as a discrete measure is conventionally the radial projection of net motion away from a given point. In contrast, the analyses here separated the two components of movement towards (convergence) and away from (divergence) each point. Furthermore, convergence and divergence fields were formed from summing the total distances ridden in any direction—not just the radial component—oriented respectively towards and away from each point. Radial flux represents the degree to which cultural geography promotes movement directly towards (for convergence) or away from (for divergence) particular places, while the full flux fields analysed here encapsulate both this sense of direct movement, along with the degree to which places of cultural or geographical significance are also places around which movement circles in not necessarily direct patterns of convergence and divergence. Equivalent relationships with radially projected measures of convergence and divergence were qualitatively identical, with merely weakened strengths of relationship, and are presented in Fig. 2C and Table 4.

**Table 4:** Repeat of Table 2, but using radial convergence and divergence, rather than the full measures as explained above.

Variables	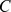	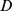	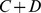	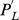
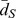	0.14	**0.31**	0.31	
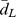	−0.12	−0.24	**−0.25**	
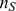	0.31	0.13	**0.31**	0.17
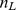		**0.43**	0.29	
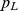	−0.36			**0.60**
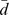	**−0.36**		−0.18	0.39
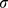				
		**−0.36**	−0.23	

Values are strengths of relationship (as 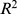 correlation coefficients) between divergence (D), convergence (C), total flux (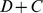), and 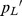 modelled from C and D as explained in main text; and lengths and numbers of short and long trips, the proportion of long trips, and mean (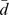) and SD (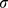) distance. Bold font indicates maximal correlation for each movement variable. Values of 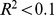 are not shown.

**Figure 8:**
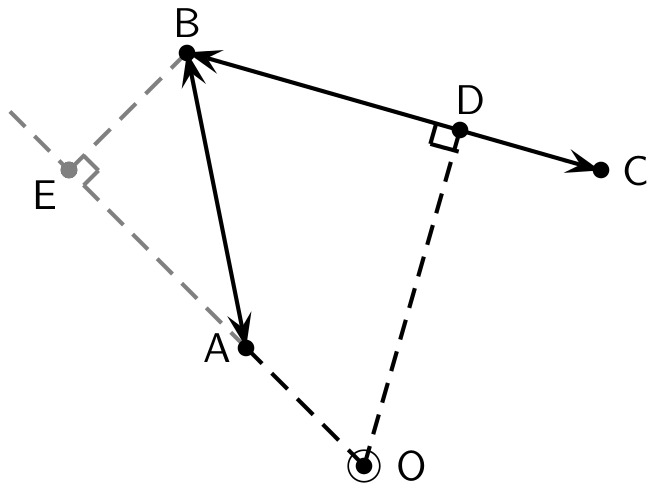
Calculation of convergence and divergence. Values measured with reference to the point ‘O’.

The calculation is illustrated in Fig. 8, for which the trip 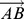 is directed away from 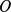, and therefore adds the distance 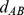 to the divergence field at 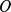, weighted by the distance to the nearer station, 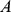, according to 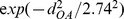. The trip 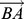 adds the same value of 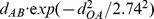 to the convergence field at 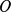. The trip 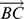, which is intersected by the perpendicular to 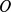, adds 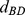 to the convergence field and 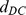 to the divergence field, both weighted according to the distance to the closest point, which is to the orthogonal intersection, 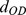. The trip 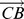 simply reverses these contributions, such that 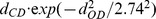 is added to the convergence field, and 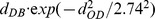 is added to divergence.

Values of radial flux are such that the trip 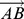 contributes the component 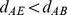, again weighted by distance to the nearest point, 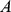. Thus, 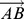 adds 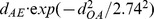 to the divergence field at 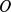, while 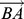 adds the same value to the convergence field. The trips 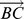 and 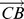 do not contribute to radial divergence.

### Simulation of neutral values of convergence and divergence

Convergence and divergence calculated as described above depend both on absolute numbers of trips and on directions. In the absence of any spatial structure, travel would be expected to be randomly oriented, in which case the flux fields would scale directly with numbers of rides alone. The contribution of such non-directed travel to the observed flux fields was estimated through simulating a set of rides with the same statistical properties as the observed rides, yet randomly oriented in space. Subtracting these fields from the observed fields according to Eq. 3 produces measures of convergence and divergence that exclusively reflect active orientation towards, away from, or around points of cultural or geographic significance.

Flux fields expected with random orientation were calculated from a simulated set of 100 times the 1,425,884 observed trips trips, generated according to the same frequency distribution of distances (Fig. 1A) by randomly selecting a starting station along with a trip distance from the observed distribution. The end station was then chosen as the station closest in distance from the random start station to the selected distance. Because this procedure generates trips statistically likely to be directed toward the centre of the city where there are more stations, one half of all simulated trips were randomly reversed in direction. After dividing by 100, these neutral fields were subtracted from the observed convergence and divergence fields, to generate the anomaly fields presented in the main text (and in Figs. S1 and S2).

### Supporting Information – Hourly Flux Fields

To illustrate the utility of the convergence and divergence analyses in capturing the ‘daily pulse’ [28] of the city, hourly values of each field are shown in Figures S1 and S2, respectively.

## Supporting Information

Figure S1 **Hourly patterns of convergence.** Hour is indicated in lower right corner of each panel, with red lines delineating positive from negative net flux (that is, anomalies above neutral values, on scale of hundreds of ridden kilometers per hour). Longitudinal and latitudinal scales are the same as for all maps in main text, with colour scale shown on right in units of hundreds of cycled kilometres per hour.
(EPS)

Figure S2 **Hourly patterns of divergence.** Colour scale and all other aspects are precisely the same as for convergence in Fig. S1, and may be directly compared.
(EPS)
